# Delay of initial radioactive iodine therapy beyond 3 months has no effect on clinical responses and overall survival in patients with thyroid carcinoma: A cohort study and a meta‐analysis

**DOI:** 10.1002/cam4.4607

**Published:** 2022-02-18

**Authors:** Fang Cheng, Juan Xiao, Fengyan Huang, Chunchun Shao, Shouluan Ding, Canhua Yun, Hongying Jia

**Affiliations:** ^1^ Department of Epidemiology and Health Statistics, School of Public Health Cheeloo College of Medicine, Shandong University Jinan People's Republic of China; ^2^ Center of Evidence‐Based Medicine, the Second Hospital Cheeloo College of Medicine, Shandong University Jinan China; ^3^ Department of Nuclear Medicine, the Second Hospital Cheeloo College of Medicine, Shandong University Jinan China

**Keywords:** cohort study, differentiated thyroid cancer, radioactive iodine, timing of therapy

## Abstract

**Background:**

More than a third of thyroid carcinoma (TC) patients require treatment with radioactive iodine (RAI), but the timing of initial RAI therapy after thyroidectomy remains controversial.

**Methods:**

We included 1224 differentiated thyroid carcinoma (DTC) patients during 2015–2019, divided them into the early (≤3 months) and the delayed (>3 months) groups based on the interval between surgery and the initial RAI. Clinical outcomes were assessed within 6–8 months of treatment with RAI, including excellent response (ER), indeterminate response (IDR), biochemical incomplete (BIR) and structural incomplete response (SIR). Further transformed them into dichotomous outcomes, we therefore introduced the ordered/binary logistic regression to assess the relation of time interval and quaternary/dichotomous outcomes, respectively. Finally, we conducted a meta‐analysis for cohort study to investigate the effect of timing of RAI therapy on the prognosis of TC.

**Results:**

Delay RAI therapy beyond 3 months reduced the IR (BIR + SIR) rate in the present cohort study (*RR* = 0.67, 95% CI: 0.49–91). Following meta‐analysis including 38,688 DTC patients confirmed these results (*RR* = 0.77, 95% CI: 0.66–0.91), further revealed the duration of treatment does not influence OS (pooled *RR* = 1.05, 95% CI: 0.83–1.33).

**Conclusion:**

Delayed initial RAI therapy beyond 3 months but no later than 6 months did not impair the prognosis of TC.

## Graphical abstract

We conducted a cohort study to investigate whether the time of initial radioactive iodine (RAI) administration would affect the clinical responses, and incorporate these results into the current body of evidence by performing meta‐analysis. Providing a solid theoretical basis for the revision of clinical guidelines.

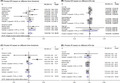

## 1 INTRODUCTION

Thyroid cancer (TC), as the most common endocrine malignancy,^1^ accounts for 3%–4% of all cancers,^2^ and approximately 90% of TC is differentiated thyroid cancer (DTC),^3^ After appropriate treatment, DTC patients carry an overall excellent prognosis, with a 5‐year survival of 98.3% and 10‐year survival of 95%.^4^ The main treatment for DTC is surgery combined with radioactive iodine (RAI) therapy. Firstly, for patients with DTC confirmed by preoperative fine needle biopsy, according to the scope of the lesion and whether it is metastatic, patients would be performed a total thyroidectomy or lobectomy, with or without lymph node dissection. Then, according to the 2015 ATA guidelines, DTC patients were stratified into different recurrence risks based on postoperative pathology, biochemical indices, and imaging findings. For patients with high recurrence risk, RAI is routinely recommended if one of the following conditions is met: DTC >4 cm, extrathyroidal or extranodal extension, or distant metastatic disease. For patients with intermediate recurrence risk, the guidelines on RAI therapy is less clear due to conflicting evidence on the benefit of recurrence. In addition, for patients with low recurrence risk, especially whose tumor size <1 cm, any dose of RAI therapy would not be recommended.^8^

An appropriate postoperative RAI therapy can be used to clear the nail or lesion,^5^ helping to reduce disease recurrence and thyroid cancer‐related mortality.^6^, ^7^ In the past decade, in addition to the opened question of “whether and whom to treat” in postoperative RAI treatment, scholars have begun to pay attention to another clinical question, that DTC patients with an excellent prognosis and a relatively wide treatment window, when should they receive RAI therapy, does delay or early treatment affect prognosis^9^, ^10^, ^11^, ^12^, ^13^, ^14^, ^15^, ^16^, ^17^, ^18^, ^19^? Indeed, the importance of timing of initial postoperative RAI is often overlooked, even the 2015 ATA guidelines^8^ did not recommend an adequate treatment duration for RAI. Early treatment with RAI after surgery, if necessary, has a significant benefit in reducing the recurrence rate and improving survival. However according to the guidelines,^8^ whether to receive RAI should be determined by the post‐operative biochemical evaluation and neck ultrasound (US) evaluation performed 3–4 months after surgery,^20^ until then RAI treatment cannot be determined. Previously studies suggested 3 months after thyroidectomy is generally considered adequate for RAI administration.^13^ While DTC is known as with a low risk of death and a long treatment window, the specific treatment differs from man to man, which may be influenced by the patient's physical condition, social‐environmental, treatment willingness and disease‐related factors.^14^ Several studies have shown no difference in short clinical responses or overall survival (OS) between early and delayed RAI.^15^, ^16^, ^17^, ^18^ While other studies advocated that a delayed RAI may cause poor OS for DTC patients after 5 or 10 years follow‐up compared with early RAI therapy,^9^, ^21^ or claimed that applying RAI therapy within 3 months could decrease ablation success.^9^

Based on the above contradictory and one‐sided reports, we therefore conducted a cohort study to illustrate whether early or delayed treatment of RAI affects the prognosis of DTC patients, and incorporate these results into the current body of evidence by performing a meta‐analysis.

## 2 MATERIALS AND METHODS

### 2.1 Study population

3455 DTC patients who received RAI therapy were collected in our department (Department of Nuclear Medicine, the Second Hospital of Shandong University, Jinan, Shandong province, China) from December 2015 to May 2019. Inclusion criteria are as follow: (1) received total or near‐total thyroidectomy; (2) the pathological diagnosis was DTC; (3) keep a low‐iodine diet and thyroid hormone withdrawal 1 month before RAI therapy, until the thyroid stimulating hormone (TSH) exceeded 30 μIU/ml; (4) clinical response was assessed at 6–8 months after initial RAI therapy. There are several exclusion criteria: (1) with a history of an RAI therapy (*n* = 1367); (2) lack of detailed case data for surgery (*n* = 645); (3) without low iodine diet (*n* = 67); (4) lost follow‐up after RAI therapy (*n* = 152). Finally, 1224 eligible DTC patients were included.

### 2.2 RAI therapy and follow‐up protocol

The therapeutic dose of iodine‐131 ranged from 3.00 GBq (80 mCi) to 5.55 GBq (150 mCi). Our study included some patients for whom RAI therapy was not routinely recommended due to the following special circumstances.^8^ Some low‐risk patients receive RAI for the reason of invasion of tumor into the perithyroidal soft tissues and personal wishes, and some intermediate‐risk patients received RAI treatment because of vascular aggression or aggressive histology (e.g., tall cell, hobnail variant, columnar cell carcinoma). Patients were hospitalized for 3 days after receiving RAI therapy, given levothyroxine regularly, and were regularly followed up by physical examination, biochemical indicators, serum thyroglobulin (Tg), serum anti‐Tg antibody (TgAb), and neck ultrasound (US). Other examinations are performed selectively based on the patient's wishes.

### 2.3 Data collection

According to previous literature,^13^ the period ≤3 months was termed as early group, otherwise as delay group. Based on the results of biochemical and imaging examinations after 6–8 months of RAI treatment, we classified patients into the following four groups: excellent response (ER), indeterminate response (IDR), biochemical incomplete (BIR) and structural incomplete response (SIR).^8^ The quaternary outcomes were further transformed into dichotomous outcomes, including ER/ non‐ER (including IDR, SIR and BIR), and IR (BIR + SIR)/ non‐IR. The determination methods of laboratory related indicators including TSH, Tg and TgAb, have been reported in previous works of literature.^22^ Neck ultrasound was performed using a real‐time ultrasound system (LOGIQ E9; General Electric Healthcare) with a 9‐ to 15‐MHz linear probe. An e.cam spect (SIEMENS, Germany) with high‐energy collimators was equipped to perform ^131^I imaging, and the scan speed was 12 cm/min with total counts of at least 140.000 cpm. Other related information was obtained from the electronic medical record system. Tumor‐node‐metastasis (TNM) stage was evaluated according to the 8th edition of the AJCC/TNM,^23^ and evidence for recurrence risk stratification comes from the 2015 guidelines.^8^

### 2.4 Statistical analysis

STATA version 16 (Stata Corporation) was the software for all statistical analysis in the present study. All reported *P*‐value were two‐sided, and a significant level was set as *p* < 0.05. Data were expressed as mean ± standard deviation (SD) or frequency (percentage). In the correlation analysis, continuous variables were tested by one‐way analysis of variance (ANOVA) or the Kruskal–Wallis test, and categorical variables were checked by the Cochran–Mantel–Haenszel *χ* ^2^ test. The analytical procedures employed were ordered logistic regression to evaluate the relative risk (*RR*) of the results of four categories, and a binary logistic regression to assess *RR* for dichotomous outcomes.

### 2.5 Meta‐analysis

**FIGURE 1:**
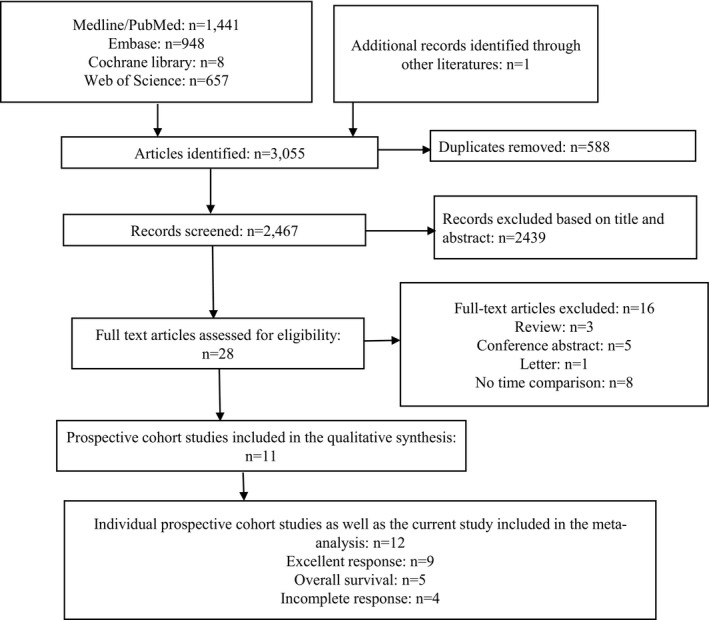
The flow chart of searching and selecting literature

As of February 20, 2021, we have systematically searched the following five commonly used electronic resource databases, including PubMed, Embase, Cochrane Library and Web of Science, and the detailed retrieval strategies were shown in Table S1. Based on the detailed guidelines of Preferred Reporting Items for Systematic Reviews and Meta‐Analyses (PRISMA),^24^ we identified 11 prospective cohort studies with 37,464 participants,^9^, ^10^, ^11^, ^12^, ^13^, ^14^, ^15^, ^16^, ^17^, ^18^, ^19^ and the flow chart of literature filtering step by step was shown in Figure 1. The quality of each study was assessed by the Newcastle‐Ottawa quality scale (NOS),^25^ and ≥7 as considered of high quality. Primary endpoints were ER (Met the following conditions: Negative imaging, and Suppressed Tg <0.2 ng/ml or TSH stimulated Tg <1.0 ng/ml), IR (BIR + SIR) and OS. The number of IR and ER cases and the total population were collected to calculate the combined *RRs*. The hazard ratio (*HR*) and 95% confidence intervals (95% CI) extracted from the multivariate analysis were collected to calculate the correlation between OS and treatment time, otherwise, the *HRs* and 95% CI of the univariate analysis were utilized. The calculation of pooled‐meta effect was based on the following methods: the inverse variance weighted mean of the logarithm of *RR* or *HR* with 95% CI. Heterogeneity within the group was assessed by *I* ^2^ statistic,^26^ if *I* ^2^ ≥ 50%, we would introduce a random‐effects model to calculate the summary estimates,^27^ otherwise, we would adopt a fixed effect model.^28^

## 3 RESULTS

### 3.1 Characteristics of the included patients in the cohort

Table 1 showed 830 patients were assigned to the early group (≤3 months from surgery to initial RAI therapy) and the other 394 patients to the delayed group (>3 months from surgery to initial RAI therapy). The average age of the patients at the time of surgery was 43.44 years old, about 71.08% were female, 42.73% of patients were in T1a, 38.73% of patients had lymph node metastases, and 75.74% of patients were at intermediate risk, only about 10% of the patients with low risk. All the variables involved in this study were evenly distributed between the early and delay group, so no additional propensity score matching or subgroup analysis was required.

**TABLE 1:** Clinical characteristics according to the timing of radioactive iodine therapy

	Total	Early group	Delay group	*Z*/*χ* ^2^	*p*
Number of patients	1224	830	394		
Age (year)	43.44 (±12.08)	43.14 (±12.44)	44.09 (±11.32)	1.53	0.22^b^
Sex (female)	870 (71.08)	578 (69.64)	292 (74.11)	2.01	0.11^a^
Tumor number	2.85 (±9.79)	2.77 (±9.45)	3.02 (±10.49)	1.04	0.31^c^
Max tumor size (cm)	1.40 (±1.02)	1.42 (±1.02)	1.36 (±1.02)	0.70	0.40^b^
Tumor stage					
T_0_	3 (0.25)	1 (0.12)	2 (0.51)	7.39	0.39^a^
T_1a_	523 (42.73)	342 (41.20)	181 (45.94)		
T_1b_	382 (31.21)	273 (32.89)	109 (27.66)		
T_2_	158 (12.91)	108 (13.01)	50 (12.69)		
T_3a_	17 (1.39)	13 (1.57)	4 (1.02)		
T_3b_	23 (1.88)	17 (2.05)	6 (1.52)		
T_4a_	4 (0.33)	2 (0.24)	2 (0.51)		
T_x_	114 (9.31)	74 (8.92)	40 (10.15)		
Node stage					
N_0_	152 (12.42)	100 (12.05)	52 (13.20)	1.98	0.37^a^
N_1a_	598 (48.86)	417 (50.24)	181 (45.94)		
N_1b_	474 (38.73)	313 (37.71)	161 (40.86)		
Distant metastases	5 (0.41)	5 (0.60)	0 (0.00)	2.38	0.12^a^
TNM stage					
I	1032 (84.31)	697 (83.98)	335 (85.03)	0.28	0.87^a^
II	188 (15.36)	130 (15.66)	58 (14.72)		
III	4 (0.33)	3 (0.36)	1 (0.25)		
Recurrence risk^*^					
Low	125 (10.21)	81 (9.76)	44 (11.17)	0.82	0.66^a^
Intermediate	927 (75.74)	629 (75.78)	298 (75.63)		
High	172 (14.05)	120 (14.46)	52 (13.20)		
Thyroid operation					
Total	1088 (88.89)	729 (88.83)	359 (91.12)	2.92	0.08^a^
Near‐total	136 (11.11)	101 (12.17)	35 (8.88)		
Lymph node dissection				0.03	0.84^a^
Lateral and central	458 (39.62)	309 (37.22)	149 (37.82)		
Central	766 (60.38)	521 (62.78)	245 (62.18)		
Dose of I^131^(mCi)	109.2 (110–110)	107.6 (110–110)	110.2 (110–110)	1.10	0.27^b^
Pre‐ablative Tg (μg/L)	2.53 (0.55–9.40)	2.40 (0.52–8.80)	2.80 (0.60–10.28)	0.80	0.37^c^

Early group: time interval ≤3 months; Delay Group: time interval >3 months.

Unless indicated otherwise, continuous data are given as the mean (±SD) for normal distribution or median (*P*_*25*_ *–P*_*75*_); categorical data were used as frequencies (%).

^a^ Cochran–Mantel–Haenszel χ^2^ test.

^b^ One‐way analysis of variance.

^c^ Kruskal‐Wallis test.

After receiving RAI treatment, patients experienced a median follow‐up of 7.2 months, and the follow‐up time did not differ between the two groups. The distribution of clinical outcomes significantly differentiated between groups (Early group: ER (49.40%), IDR (14.34%), BIR (8.19%) and SIR (28.07%); Delay group: ER (51.78%), IDR (15.69%), BIR (9.39%) and SIR (20.30%). *χ* ^2^ = 10.04, *p* = 0.02). When we further combined them into dichotomous outcomes, the distribution of ER in the early and delay group were 49.40% and 51.78% without statistical difference. However, the rates of IR were not evenly distributed between the two groups (Early group: 36.27%; Delay group: 29.70%. *χ* ^2^ = 5.13, *p* = 0.024) (Table 2).

**TABLE 2:** The distribution of clinical responses among the early and delayed group

	Total	Early group	Delay group	*Z*/*χ* ^2^	*p*
Number of patients	1224	830	394		
Therapy time interval (day)	79 (63–97)	69 (60–80)	112 (99–144)	801.87	**<0.001** ^b^
Time of evaluating outcomes (month)	7.2 (6.52–8.14)	7.1 (6.23–8.20)	7.4 (6.86–7.92)	2.34	0.12^b^
Quaternary outcomes					
ER	614 (50.16)	410 (49.40)	204 (51.78)	10.04	**0.02** ^a^
IDR	192 (15.69)	119 (14.34)	73 (18.53)		
BIR	105 (8.58)	68 (8.19)	37 (9.39)		
SIR	313 (25.57)	233 (28.07)	80 (20.30)		
Dichotomous outcomes based on ER					
ER	614 (50.16)	410 (49.40)	204 (51.78)	0.64	0.437^a^
(IDR + BIR + SIR)	610 (49.84)	420 (50.60)	190 (48.22)		
Dichotomous outcomes based on IR					
IR (BIR + SIR)	418 (34.15)	301 (36.27)	117 (29.70)	5.13	**0.024** ^a^
(ER + IDR)	806 (65.85)	529 (63.73)	277 (70.30)		

Early group: time interval ≤3 months; Delay Group: time interval >3 months.

Categorical data were used as frequencies (%); therapy time interval is given as median (*P*_*25*_ *–P*_*75*_).

Abbreviations: BIR, biochemical incomplete response; ER, excellent response; IDR, indeterminate response; IR, incomplete response; SIR, structural incomplete response.

^a^ Cochran–Mantel–Haenszel *χ* ^2^ test.

^b^ Kruskal‐Wallis test.

### 3.2 Factors associated with clinical outcomes of RAI therapy

Univariate ordered logistic regression showed that the gender of women (*RR* = 0.51, *p* < 0.001) was a protective factor for clinical outcomes (Table 3). All other variables were risk factors, including age >55 years old (*RR* = 1.48, *p* < 0.01), number of tumors >2 (*RR* = 1.42, *p* < 0.01), max tumor size >2 cm (*RR* = 1.64, *p* < 0.01), high‐risk of recurrence (*RR* = 6.61, *p* < 0.001), central lymph node dissection only (*RR* = 1.57, *p* < 0.001), and pre‐ablative Tg level >2 ng/ml (*RR* = 4.06, *p* < 0.001). In multiple ordered logistic regression, patients who received a delayed RAI were 24% (95% CI: 4%–42%, *p* = 0.02) less likely to be classified as IDR, BIR or SIR. For dichotomous outcomes (IR/[ER + IDR]), the binary logistic regression (Table 4) found that after adjusting the possible confounding factors, a treatment interval of more than 3 months could result in a 33% (95% CI: 9%–51%, *p* = 0.01) decrease in IR.

**TABLE 3:** Univariate and multivariable ordered logistic regression to determine related factors in relation to clinical outcomes of RAI therapy

	Univariate			Multivariable		
	*RR*	95% CI	*p*	*RR*	95% CI	*p*
Age (Ref: ≤55)						
>55	1.48	(0.12,1.94)	<0.01	1.47	(1.06,2.02)	0.02
Gender (Ref: male)						
Female	0.51	(0.40,0.64)	<0.001	0.57	(0.44,0.74)	<0.001
Tumor number (Ref: ≤1)						
1–2	1.02	(0.78,1.33)	0.89	1.09	(0.82,1.44)	0.57
>2	1.42	(1.11,1.82)	<0.01	1.11	(0.83,1.49)	0.46
Max tumor size (Ref: ≤1 cm)						
1–2 cm	1.06	(0.83,1.36)	0.64	0.90	(0.69,1.18)	0.45
>2 cm	1.64	(1.20,2.23)	<0.01	0.96	(0.69,1.34)	0.82
TNM stage (Ref: I)				NA		
II	1.30	(0.98,1.73)	0.07			
III	7.42	(0.74,73.95)	0.08			
Recurrence risk (Ref: low)						
Intermediate	1.11	(0.76,1.60)	0.594	1.08	(0.71,1.63)	0.71
High	6.61	(4.22,10.37)	<0.001	4.23	(2.54,7.04)	<0.001
Lymph node dissection (Ref: lateral and central)						
Central	1.57	(1.25,1.96)	<0.001	1.47	(1.15,1.89)	<0.01
Pre‐ablative Tg (Ref: <2 ng/ml)						
≥2 ng/ml	4.06	(3.12,5.13)	<0.001	3.07	(2.36,3.99)	<0.001
Therapy time (Ref: ≤3 month)						
>3 month	0.82	(0.65,1.02)	0.08	0.74	(0.58,0.96)	0.02

Abbreviations: 95% CI, 95% confidence interval; NA, the present factor was not included in the multivariate analysis; Ref.: Reference group; *RR*, relative risk.

**TABLE 4:** Logistic regression with the timing of RAI therapy and different clinical outcomes

Clinical outcome	Univariate			Multivariable		
	*RR*	95% CI	*p*	*RR*	95% CI	*p*
ER/(IDR + BIR + SIR)	1.10	(0.87, 1.40)	0.44	1.21	(0.91, 1.26)	0.19
IR/(ER + IDR)	0.74	(0.57, 0.96)	0.02	0.67	(0.49, 0.91)	0.01

*Note*: The confounding variables adjusted by multivariate logistic regression including: age, gender, number of the tumor, size of the tumor, ATA risk stratification, Location of prophylactic lymph node dissection and level of Pre‐ablative Tg.

Early group (time interval ≤3 months) as the control group; Delay Group (time interval >3 months).

Abbreviations: 95% CI, 95% confidence interval; BIR, biochemical incomplete response; ER, excellent response; IDR, indeterminate response; ; IR, incomplete response; *RR*, relative risk; SIR, structural incomplete response.

### 3.3 Meta‐analysis of prospective cohort studies including the present study

Including the present study, 12 prospective studies with 38,688 participants^9^, ^10^, ^11^, ^12^, ^13^, ^14^, ^15^, ^16^, ^17^, ^18^, ^19^ were eligible for the meta‐analysis, the average score of NOS was 7, indicating these included studies in the meta‐analysis were of high quality, more details were contained in Table S2. Among them, Table 5 displayed that nine studies took ≤3 months as early treatment group,^10^, ^12^, ^13^, ^14^, ^15^, ^16^, ^17^, ^19^ one of which was grouped at ≤4.7 months,^10^ and we combined it with 3 months subgroup based on its' median closely to 3 months. The other 3 studies were taken ≤6 months as the early group.^9^, ^11^, ^18^ In terms of clinical outcomes, four studies reported the rate of OS, nine studies reported ER rates, and five studies reported IDR, BIR, and SIR.

**TABLE 5:** Clinical response and overall survival at different timing of RAI therapy in the meta‐analysis

ID	OS	ER		IDR		BIR		SIR	
	HR (95% CI)	Early	Delay	Early	Delay	Early	Delay	Early	Delay
Time interval ≤3 month as control group									
Özhan‐2021	N.R	80/116	241/294	N.R.	N.R.	N.R.	N.R.	N.R.	N.R.
Jonghwa‐2020	N.R	348/451	60/75	93/451	14/75	7/451	0/75	3/451	1/75
Mijin‐2019	1.50 (0.6–3.4)	337/556	204/360	116/556	73/360	67/556	59/360	36/556	24/360
Wang‐2019	N.R	113/199	127/200	N.R.	N.R.	N.R.	N.R.	N.R.	N.R.
Li‐2018	N.R	146/187	30/48	33/187	9/48	6/187	5/48	2/187	4/48
Suman‐2016_9	0.98 (0.71–1.34)	N.R.	N.R.	N.R.	N.R.	N.R.	N.R.	N.R.	N.R.
Suman‐2016_7	1.09 (0.75–1.58)	N.R.	N.R.	N.R.	N.R.	N.R.	N.R.	N.R.	N.R.
Tsirona‐2014	N.R	44/50	52/57	N.R.	N.R.	N.R.	N.R.	N.R.	N.R.
Our result	N.R	410/830	204/394	119/830	73/394	68/830	37/394	233/830	80/394
Time interval ≤6 month as control group									
Matrone‐2020	N.R	87/181	125/226	55/181	61/226	19/181	20/226	20/181	20/226
Scheffel‐2016	N.R	175/295	164/250	N.R.	N.R.	N.R.	N.R.	N.R.	N.R.
Higashi‐2011	0.24 (0.09, 0.65)	N.R.	N.R.	N.R.	N.R.	N.R.	N.R.	N.R.	N.R.

Abbreviations: BIR, biochemical incomplete response; CI, confidence interval; ER, excellent response; HR, hazard ratio; IDR, indeterminate response; N.R., not report; OS, overall survival; SIR, structural incomplete response.

**FIGURE 2:**
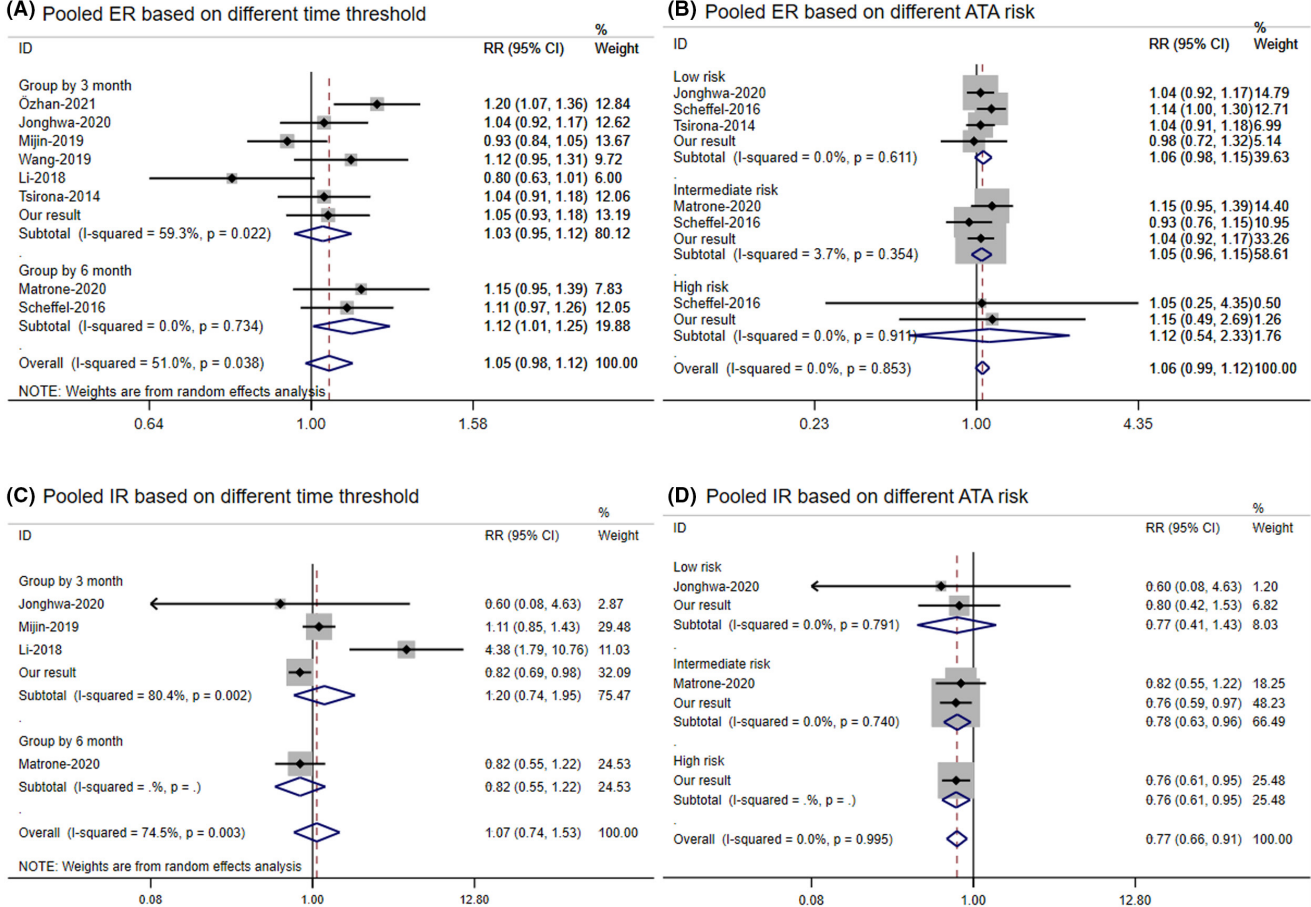
Forest plot of short clinical outcomes. (A) Pooled ER results based on different definitions of the early group; (B) Pooled ER results based on different ATA risk factors; (C) Pooled IR results based on different definitions of the early group; (D) Pooled IR results based on the different ATA risk factor. Grouped by 3 months: all involved studies taken ≤3 months as the early group. Grouped by 6 months: all involved studies taken ≤6 months as the early group. Low risk: low risk of recurrence. Intermediate risk: intermediate risk of recurrence. High risk: high risk of recurrence

Figure 2 was the pooled forest plot of short clinical responses, and Figure 2A suggested no significant effect of treatment interval on ER, with a slight heterogeneity (pooled *RR* = 1.05, 95% CI: 0.98–1.12; *I* ^2^ = 51.0%). Further stratified by time interval, delayed treatment of more than 6 months was a protective factor for ER (pooled *RR* = 1.12, 95% CI: 1.01–1.25; *I* ^2^ = 0.00%), while not significant in 3 months subgroup. Figure 2B did not support a delay of RAI therapy will affect ER rate (pooled *RR* = 1.06, 95% CI: 0.99–1.12, *I* ^2^ = 0.00%), regardless of the patient's risk stratification were low‐risk (pooled *RR* = 1.06, 95% CI: 0.98–1.15), medium‐risk (pooled *RR* = 1.05, 95% CI: 0.96–1.15), or high‐risk (pooled *RR* = 1.12, 95% CI: 0.54–2.23). Figure 2C illustrated that delayed RAI treatment did not have any effect on clinical IR rates whether at less than 3 or 6 months as the early group (pooled *RR* = 1.07, 95% CI: 0.74–1.53; *I* ^2^ = 74.5%, *p* = 0.003). Pooled IR result in Figure 2D showed that a delayed treatment slightly reduced the incidence of IR (*RR* = 0.77, 95% CI: 0.66–0.91) with excellent consistency (*I* ^2^ = 0.00%, *p* = 0.995), especially in patients with intermediate (*RR* = 0.78, 95% CI: 0.63–0.96; *I* ^2^ = 0.0%) or high (pooled *RR* = 0.76, 95% CI: 0.61–0.95) risk of recurrence.

**FIGURE 3:**
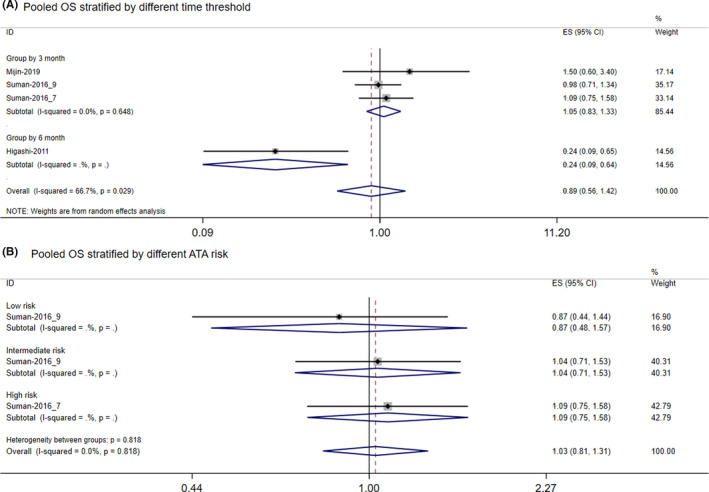
Forest plot of long‐term overall survival (OS). (A) Pooled OS results based on different definitions of the early group. (B) Pooled OS results based on the different ATA risk factors. Grouped by 3 months: all involved studies taken ≤3 months as the early group. Grouped by 6 months: all involved studies taken ≤6 months as the early group. Low risk: low risk of recurrence. Intermediate risk: intermediate risk of recurrence. High risk: high risk of recurrence

Figure 3 drew the forest plot of the long‐term OS, delayed RAI treatment beyond 3 months did not have any effect on OS rates without any heterogeneous (*I* ^2^ = 0.00%, *p* = 0.648, Figure 3A). However, one study reported a delayed treatment surpassing 6 months might reduce the OS of DTC patients (*RR* = 0.24, 95% CI: 0.09–0.65, Figure 3A). Thus, when we pooled all studies reporting OS, the pooled results showed moderate heterogeneity (*I* ^2^ = 66.7%, *p* = 0.029). Besides, there was only one study per risk stratification that showed a delayed RAI treatment did not affect OS without any heterogeneous (*RR* = 1.03, 95% CI: 0.81–1.31; *I* ^2^ = 0.0%, Figure 3B).

## 4 DISCUSSION

Applying postoperative RAI therapy after 3 months decreases IR in patients with DTC, and the pooled results of three studies support that delayed treatment is associated with lower IR rates. Meanwhile, our results and the meta‐analysis results confirm that delayed treatment, whether at 3 or 6 months before treatment with RAI, does not affect ER rates or long‐term OS.

The concentration of iodine in vivo at the time of RAI is an important factor affecting the absorption of radioactive iodine. To achieve the purpose of a focal clearing, patients were always asked to begin a low‐iodine diet 1 month before RAI treatment, combined with thyroid hormone withdrawal and other methods to reduce iodine levels in vivo, to ensure that patients can absorb as much radioactive iodine as possible during RAI treatment. As far as we know, povidone iodine‐based disinfectants are widely used for preoperative disinfection of patients, so patients undergoing thyroidectomy may absorb large amounts of iodine transdermal, these iodine would remain in the body for a short period.^29^ When the initial RAI therapy interval is delayed over 3 months, the iodine infiltrated through the skin has a higher clearance rate. In addition, the thyroid gland and its surrounding tissues would inevitably be destroyed in the duration of thyroidectomy, even causing local tissue inflammation, necrosis, and producing hematoma. In the process of postoperative local tissue recovery and reconstruction of blood supply, we believe that a longer interval can be better to restore the blood circulation and increase the dose of iodine‐131 to the residual cancer tissue.

Consistently, the meta‐analysis suggests a delayed treatment may decrease the IR rate, especially in moderate or high‐risk subgroups. However, the pooled results of two studies^11^, ^18^ suggested that a deferral of treatment beyond 6 months helped to increase the rate of ER. We systematically reviewed the characteristics of patients included in these two articles and concluded that the pooled results should be interpreted carefully. Since patients who received RAI after 6 months had one or more of the following characteristics: smaller tumor size, less percentage of lymph node metastases, less capsule invasion, lower levels Tg, older age, a higher percentage of women, T1a patients, and low‐risk patients. These characteristics suggested that patients who received a delayed RAI treatment might have a milder illness than those receiving treatment within 6 months, although these factors were evenly distributed in the present cohort study.

Further, the meta‐analysis demonstrated a delayed RAI treatment beyond 3 months does not affect long‐term OS, we suggest any initial RAI therapy should not be delayed beyond 6 months. Since a retrospective cohort study of 198 patients with metastatic DTC, showed a 3.22‐fold increased risk of death once the RAI treatment was delayed beyond 6 months.^9^ Probably because of latent progression of metastatic disease during the prolonged interval, a delayed RAI may result in poor accumulation of radioiodine at initial RAI. Besides, Jolanta et al.^21^ emphasized that DTC patients receiving initial RAI treatment delayed beyond 9 months may have a poorer long‐term prognosis, even though they have a low risk of recurrence. Due to the availability of medical resources in Japan, about half of the patients in their study received RAI for the first time between 9 and 24 months, which was significantly different from other studies that we included in the meta‐analysis. We therefore discuss their results separately.

Of course, there are several conditions we should be aware of when we delay the patient's first RAI therapy in the actual medical practice. Firstly, age is already well known as an independent risk factor,,^30^, ^31^, ^32^ and in the 8th guidelines,^23^ the prognosis is likely to be worse for patients over 55 years of age. In the present meta‐analysis, the mean age in all involved studies was under 55 years, pooled results of the present study may not be representative of all middle‐aged and elderly people, especially those significantly older than 55 years of age. Secondly, DTC is a stable tumor with a 5‐years or 10‐year OS rate of >85% from the first diagnosis, it is far from enough in just a few years to assess the development of the tumor. Only two studies^12^, ^13^ followed‐up for more than 10 years in our meta‐analysis. Thirdly, the mortality of DTC is about 0.37/100,000,^33^ more attention should be paid to the side effects caused by DTC, such as recurrence, metastasis and other complications. Lastly, BRAFV600E mutation is highly correlated with impaired iodine metabolism,^34^ and the necessity for RAI treatment in V600EBRAF‐mutated patients is still being explored. No studies included in this meta‐analysis explored the effect of V600EBRAF‐mutation, which may be the focus of future research.

This is the first high‐level comprehensive evidence to explore the association between treatment duration and TC prognosis. There are several advantages. Firstly, all data related to the cohort study were obtained from a standardized electronic medical record system of First‐class Hospital at Grade 3, and the clinical outcomes were determined based on laboratory and imaging examination, therefore providing a shred of reliable evidence to evaluate the association. In addition, based on the cohort study, we integrated all the current evidence on the treatment time by a systemic meta‐analysis, and the highly NOS quality score (median: 7) of all involved studies indicated the results had high credibility. However, certain limitations of our study must be noted. The clinical outcomes collected in the cohort study were assessed after 6–8 months of follow‐up, we could only evaluate short‐term clinical effects, and longer observation periods may be required to determine the effect of treatment duration on recurrence, metastasis, and overall survival. Besides, we included different risk‐stratified patients in the meta‐analysis, although subgroup analysis was introduced to reduce heterogeneity, the fewer studies within per subgroup maybe not be strong enough to draw a credible conclusion. Finally, previous studies have proved that the short‐term clinical effect can better represent the long‐term survival rate, and the meta‐analysis summarized the impact of treatment time on the long‐term survival rate to supplement the deficiencies of existing studies, but we should still pay attention to the fact that the small sample size and moderate heterogeneity may bias the conclusion.

## 5 CONCLUSION

In conclusion, a delay of more than 3 months of RAI therapy did not impair short‐term clinical outcomes or long‐term overall survival for patients with DTC. Physicians can make appropriate arrangements for patients based on the availability of medical resources within a period not exceeding 6 months. We are the first to synthesize, update, and determine the adequate time of initial RAI therapy for patients who received thyroidectomy, which provides a solid theoretical basis for the revision of clinical guidelines.

## CONFLICT OF INTEREST

The authors declare that they have no conflict of interest.

## AUTHORS' CONTRIBUTIONS

F CHENG conceptualized the study, performed the statistical analyses and wrote the first manuscripts. CH Yun collected the data of the cohort study, further provided ideas for the analysis of the cohort study. Hongying JIA designed and revised the manuscript, provided statistical analysis methods and approved the final manuscript. J Xiao and FY Huang conducted the literature search and excluded duplicate papers, CC Shao and SL Ding independently reviewed the titles and abstracts of the retrieved articles. All the authors listed have approved the manuscript that is enclosed.

## ETHICS APPROVAL

The use of medical record data was approved by the Institutional Review Board of the Second Hospital of Shandong University approved the use of medical records (KYLL‐2018[LW] 013), and informed consent has been obtained from all patients at the time of the RAI therapy plan.

### CODE AVAILABILITY

All statistical analyses were performed using STATA.16 software. All related code can be obtained by contacting the corresponding author.

## Supporting information


**Appendix S1** **:** Supporting information

## Data Availability

The datasets of generated analyzed during the current study are not publicly available.
