# Supplementary material for: Delay of initial radioactive iodine therapy beyond 3 months has no effect on clinical responses and overall survival in patients with thyroid carcinoma: A cohort study and a meta‐analysis
Source: Cancer Med. 2022 Feb 18;11(12):2386–96. doi: 10.1002/cam4.4607 (PMC9189474; doi:10.1002/cam4.4607)
Supplement: Supplementary file 1 — Appendix S1 : Supporting information [file CAM4-11-2386-s001.docx]

**Table s1 Strategies and databases of literature retrieval**

| **No.** | **Database** | **Website** | **Search strategy** |
| --- | --- | --- | --- |
| 1 | PubMed | https://pubmed.ncbi.nlm.nih.gov/ | ((“Thyroid Neoplasms/radiotherapy”[Majr] AND ablat*) OR (thyroid AND (cancer OR carcinoma))) AND (radioiodine OR radiotherapy) AND ablat* AND (survival OR response OR effect OR outcome OR recurrence) |
| 2 | EMBASE | http://ovidsp.dc2.ovid.com/sp-4.02.0b/ovidweb.cgi | (thyroid AND neoplasm* AND radiotherapy AND ablat* OR (thyroid AND (cancer OR carcinoma))) AND (radioiodine OR radiotherapy) AND ablat* AND (survival OR response OR effect OR outcome OR recurrence) AND [embase]/lim |
| 3 | The Cochrane Library | https://www.cochranelibrary.com/advancedsearch | (Thyroid neoplasms or thyroid) and (cancer or carcinoma) and (radioiodine or radiotherapy) and ablat* and (survival or response or effect or outcome or recurrence) |
| 4 | Web of Science | http://isiknowledge.com/ | Ts=(("thyroid cancer" OR "thyroid carcinoma " OR "thyroid neoplasms" ) AND (iodine OR radio* OR ablat*) AND (survival OR response OR effect OR outcome OR recurrence)) |

Date: up to on February 20, 2021

**Table s2 Patients clinical characteristics and ablation criteria in the meta-analysis**

| **Author** | **Patients** | **Female** | **Age** | **Follow** | **NOS** | **Histology** | **T** | **N** | **M** | **Stage** | **Risk** | **I^131^** |
| --- | --- | --- | --- | --- | --- | --- | --- | --- | --- | --- | --- | --- |
| (year) | (*No.*) | (%) | (year) | (year) | score | (%) | (%) | (%) | (%) | (%) | (%) | (mCi) |
| Özhan-2021(9) | 503 | 417(82.9) | 47.4±12.5 | 1 | 7 | PTC: 475(94.4%) | T1: 328(65.2) | N0: 454(90.2) | M0:503(100) | N.R. | low: 388(77.1) | 100 |
|  |  |  |  |  |  | Others: 28(5.6) | T2: 112(22.2) | N1: 499(9.8) |  |  | low-inter: 115(22.9) |  |
|  |  |  |  |  |  |  | T3: 44(8.8) |  |  |  |  |  |
|  |  |  |  |  |  |  | T4A: 19(3.8) |  |  |  |  |  |
| Jonghwa-2020(11) | 526 | 433(82.3) | 47.2(39–55) | 9.1 | 8 | PTC: 526(100%) | T1: 445 (84.6) | N0:446(84.8) |  | N.R. | low: 526(100) | 30-150 |
|  |  |  |  |  |  |  | T2: 68 (12.9) | N1: 80(15.2) |  |  |  |  |
|  |  |  |  |  |  |  | T3a: 13 (2.5) |  |  |  |  |  |
| Matrone-2020(10) | 414 | 756(83) | <=55: | 6 | 7 | CV-PTC:195 (47.1) | T1a:115(27.8) | N0:12(2.9) | M0:242(58.5) | N.R. | inter: 414(100) | 30-320(77.3) |
|  |  |  | 310(74.9) |  |  | FV-PTC:44 (10.6) | T1b:146(35.3) | N1a:81(19.6) | M1:172(41.5) |  |  | 100-94 (22.7) |
|  |  |  |  |  |  | AV-PTC:158 (38.2) | T2:96 (23.2) | N1b:91(22) |  |  |  |  |
|  |  |  |  |  |  | FTC:17 (4.1) | T3:57 (13.8) | Nx:230(55.6) |  |  |  |  |
| Mijin-2019(13) | 916 | 756(83) | 47.2±12.3 | 2 | 8 | PTC:916(100) | N.R. | N0:261(28) | M0:884(97) | Ⅰ:359(39) | inter and high | 138.5±32.6 |
|  |  |  |  |  |  |  |  | N1a:408(45) | M1:32 (3) | Ⅱ:17(2) |  |  |
|  |  |  |  |  |  |  |  | N1b:247(27) |  | Ⅲ:370(40) |  |  |
| Wang-2019(12) | 399 | 278(69.7) | 44.3±13.5 | 1 | 6 | PTC:389 (97.5) | N.R. | N.R. | N.R. | Ⅰ:218 | low:87(21.8) | 113.20±38.72 |
|  |  |  |  |  |  | FTC:10(2.5) |  |  |  | Ⅱ:9(2.3) | inter:170(42.6) | |
|  |  |  |  |  |  |  |  |  |  | Ⅲ:71(17.8) | high:142(35.6) | |
|  |  |  |  |  |  |  |  |  |  | Ⅳ:101 |  |  |
| Li-2018(14) | 235 | 143(61) | 42(33-50) | 2.1 | 7 | PTC:226(96.2) | T1:120(51.1) | N0:42 (17.9) | M0:399(100) | N.R. | low and inter | 30:163(69.4) |
|  |  |  |  |  |  | FTC:9(3.8) | T2:13 (5.53) | N1a:120 (51.1) |  |  |  | 100:72(30.6) |
|  |  |  |  |  |  |  | T3:102 (43.4) | N1b:73(31.1) |  |  |  |  |
| Suman-2016(15) | 23915 | 19608 (82) | 47.1±13.3 | 10 | 7 | PTC:15120 (63) | T0-T2 | N0:23915(100) | M0=23915(100) | N.R. | low:12025(50.3) | N.R. |
|  |  |  |  |  |  | FV-PTC:8795 (37) |  |  |  |  | inter:11890(49.7) | |
|  |  |  |  |  |  |  |  |  |  |  |  |  |
| Suman-2016(16) | 9706 | 6558(68) | 45.5±15.5 | 10 | 7 | PTC:7299 (75) | T3-T4 | N0:1138 (12) | N.R. | N.R. | high | N.R. |
|  |  |  |  |  |  | FV-PTC:2146 (22) |  | N1:7926 (82) |  |  |  |  |
|  |  |  |  |  |  | AV-PTC:261 (3) |  | unknow:546 (6) |  |  |  |  |
| Scheffel-2016(17) | 545 | 436 (80.0) | 44.9±15.1 | 6 | 7 | PTC:464 (85.1) | N.R. | N0:359 (65.9) | M0:495(90.8) | Ⅰ:322(59.1) | low:228(41.8) | 111.7±36.9 |
|  |  |  |  |  |  |  |  | N1:186 (34.1) | M1:50 (9.2) | Ⅱ:62(11.4) | Inter:245(45.0) | |
|  |  |  |  |  |  |  |  |  |  | Ⅲ:65(11.9) | high:72(13.2) | |
|  |  |  |  |  |  |  |  |  |  | Ⅳ:82(15.0) |  |  |
|  |  |  |  |  |  |  |  |  |  | unknow:14(2.6) | |  |
| Tsirona-2014(18) | 107 | 87(81.3%) | 49.3±1.5 | 6.9 | 6 | PTC:100 (93.4) | N.R. | N0:107(100) | M0:107(100) | Ⅰ:107(100) | low: 107(100) | 73 |
|  |  |  |  |  |  | FTC:5(4.7) |  |  |  |  |  |  |
|  |  |  |  |  |  | P+DTC:2(1.9) |  |  |  |  |  |  |
| Higashi-2011(19) | 198 | 121(61%) | 54.0(7–84) | 5.4 | 7 | PTC:164(83) | N.R. | N0:151(76.3) | M0:47(24) | Ⅱ-Ⅳ | inter | 102(8-162) |
|  |  |  |  |  |  | FTC:28(14) |  | N1:47(23.7) | M1:151(76) |  | high |  |
|  |  |  |  |  |  | Other:6(3) |  |  |  |  |  |  |

Continuous variables are presented as means + SD or range (median), and categorical variables are presented as numbers (percentages). N.R.: not report. DTC: differentiated thyroid cancer; PTC: Papillary Thyroid Cancer; CV-PTC: Classic Variant of Papillary Thyroid Cancer; FV-PTC: Follicular Variant of Papillary Thyroid Cancer; AV-PTC: Aggressive Variant of Papillary Thyroid Cancer; TNM, tumor node metastasis; Risk stratification: Recurrence risk stratification; rhTSH: recombinant human TSH; THW: thyroxine withdrawal; total: total thyroidectomy; near: near total thyroidectomy.
